# Two Volatile Organic Compounds Trigger Plant Self-Defense against a Bacterial Pathogen and a Sucking Insect in Cucumber under Open Field Conditions

**DOI:** 10.3390/ijms14059803

**Published:** 2013-05-08

**Authors:** Geun Cheol Song, Choong-Min Ryu

**Affiliations:** 1 Molecular Phytobacteriology Laboratory, Systems and Synthetic Biology Research Center, KRIBB, Daejeon 305-806, Korea; E-Mail: song@kribb.re.kr; 2 Biosystems and Bioengineering Program, University of Science and Technology, Daejeon 305-333, Korea

**Keywords:** plant growth-promoting rhizobacteria (PGPR), induced resistance (ISR), systemic acquired resistance (SAR), salicylic acid (SA), jasmonic acid

## Abstract

Systemic acquired resistance (SAR) is a plant self-defense mechanism against a broad-range of pathogens and insect pests. Among chemical SAR triggers, plant and bacterial volatiles are promising candidates for use in pest management, as these volatiles are highly effective, inexpensive, and can be employed at relatively low concentrations compared with agrochemicals. However, such volatiles have some drawbacks, including the high evaporation rate of these compounds after application in the open field, their negative effects on plant growth, and their inconsistent levels of effectiveness. Here, we demonstrate the effectiveness of volatile organic compound (VOC)-mediated induced resistance against both the bacterial angular leaf spot pathogen, *Pseudononas syringae* pv. lachrymans, and the sucking insect aphid, *Myzus persicae*, in the open field. Using the VOCs 3-pentanol and 2-butanone where fruit yields increased gave unexpectedly, a significant increase in the number of ladybird beetles, *Coccinella septempunctata*, a natural enemy of aphids. The defense-related gene *CsLOX* was induced by VOC treatment, indicating that triggering the oxylipin pathway in response to the emission of green leaf volatiles can recruit the natural enemy of aphids. These results demonstrate that VOCs may help prevent plant disease and insect damage by eliciting induced resistance, even in open fields.

## 1. Introduction

Induced resistance is plant innate resistance to a variety of plant enemies, including insect and microbial pathogens [1]. To date, two types of induced resistance have been identified, namely, systemic acquired resistance (SAR) and induced systemic resistance (ISR). SAR was discovered by Ross, who identified plant systemic induced resistance while studying virus-plant interactions [2]. In addition, later studies revealed that SAR also occurred systemically following primary infection with a necrotizing (e.g., avirulent biotrophic) pathogen accompanied by increased levels of salicylic acid and pathogenesis-related proteins resulting in long-lasting effectiveness and development of a broad-spectrum resistance [3].Additionally, plant growth-promoting rhizobacteria (PGPR), a type of root-associated bacterium (rhizobacterium) that increases plant growth and yield under greenhouse and field conditions, elicit induced resistance referred to as ISR [4,5]. PGPR produce many bacterial determinants that induce ISR [4], including salicylic acid (SA), 2,4-diacetylphloroglucinol, and siderophores, as well as cell envelope substances, such as lipopolysaccharides and exopolysaccharides [3]. In 2004, volatile organic compounds (VOCs) emitted by two *Bacillus* spp. were also shown to be determinants of ISR [6]. Exposure of *Arabidopsis* to VOCs from *Bacillus subtilis* strain GB03 and *B. amyloliquefaciens* strain IN937a produces a significant reduction in symptom development caused by the soft-rot casual pathogen, *Pectobacterium carotovorum* subsp. *carotovorum* (syn. *Erwinia carotovora* subsp. *carotovora*), through the activation of the ethylene signaling pathway [6]. Proteomic analysis of *Arabidopsis* tissues treated with bacterial volatiles from strain GB03 further supported the role of ethylene by confirming the high expression levels of ethylene biosynthesis-related and ethylene-related defense response proteins [7]. Biochemical analysis of the bacterial volatiles in strain GB03, and mutational studies of the volatile biosynthesis genes in this strain, revealed that the C4 hydrocarbon 2,3-butanediol is the active ingredient (among more than 30 volatiles) that elicits ISR [6,8]. In addition to Gram-positive bacteria, later studies demonstrated that 2,3-butanediol from the Gram-negative bacterium *Pseudomonas chlororaphis* strain O6 also effectively protects tobacco plants against *E. carotovora* subsp. *carotovora* [9]. However, bacterial volatiles, such as 2,3-butanediol, and its precursor, acetoin, are difficult to apply to crop plants in open agricultural systems due to the rapid evaporation of these substances into the air, as well as the low efficacy of these bacterial volatiles compared with chemical triggers. Despite these issues, the application of 2,3-butanediol to tobacco seedlings was recently shown to reduce symptom development, even under field conditions [10,11].

In contrast to bacterial volatiles, chemical triggers exhibit a consistent, long-lasting effect on SAR in plants. The best example of such chemical triggers is benzothiadizole (BTH), which was commercialized by Syngenta (formerly Novartis) as Actigard^®^ in the USA, and as BION^®^ in Europe. BTH is the first commercialized SAR-inducing chemical that is effective against a broad spectrum of pathogens and insect pests in many crop species [12]. Studies of the molecular mechanisms in plants that are affected by BTH application indicated that BTH application increases endogenous SA levels, which activates SA-dependent signaling pathways in plants [13]. However, BTH still induces SAR in SA-hydrolyzing *NahG* transgenic plants, indicating that BTH can induce resistance in the absence of SA signaling [13]. Aside from its significant advantages, BTH has significant negative effects on plant growth [14,15]. This phenomenon is referred to as “allocation fitness cost” or “trade-off” [16]. The reduction in growth results from the competing metabolic demands of plant-related compound synthesis and the requirement of a substantial amount of energy for the induction of SAR [16]. For instance, BTH-treated wheat exhibits reduced growth and decreased seed production; the reduction in growth is more significant under nitrogen-limited conditions [14]. Following this discovery, similar results were also obtained in other plant model systems [14,15,17]. However, the detailed molecular and biochemical mechanisms underlying SAR have not yet been elucidated.

The VOCs 3-pentanol, a amyl alcohol (C5H11OH) with several isomers, is emitted from many microbes, plants, and insects [18–24]. This VOC has been studied by chemical ecologists, as it is an insect pheromone in *Triatoma infestans* and *T. brasiliensis* [18–20]. Insect behavior studies show that 3-pentanol plays a critical role as a pheromone in the sexual communication of *M. mutates* [21]. In addition, 3-pentanol is produced by wild *Brassica oleracea* plants in response to infestation by the caterpillars *Pieris rapae* and *Plutella xylostella* [23,24]. Plants were recently shown to produce 3-pentanol as an olfactory stimulus. Electrophysiological responses were elicited in male and female *Batocera horsfieldi* adults by 3-pentanol from among 20 headspace VOCs of two plant species, *Rosa multiflora* and *Populus deltoides* [24]. This VOC is rarely detected in microbial cultures. A second compound, 2-butanone, which is also known as methyl ethyl ketone (CH_3_C(O)CH_2_CH_3,_ MEK), is recognized by its sharp, sweet odor, similar to butterscotch. Although 2-butanone is biosynthesized by some trees, and small amounts of this compound are found in some fruits and vegetables, the majority of 2-butanone is produced by microbes [25–27]. This ketone was detected in the headspace of aerobically and anaerobically incubated soil samples, indicating that diverse soil microbes can be used as sources for 2-butanone [26]. Specifically, previous studies on the aromatic profiling of five cryneform bacteria indicated that *Brevibacterium linens* strains produce 2-butanone [25]. In addition, 2-butanone was also detected in cultures of four out of seven isolates of the thermophillic Gram-positive bacterium actinomycetes [27]. In addition to bacteria, the saprophytic fungus *Trichoderma* spp. secretes 2-butanone [28]. More recently, root-associated *Bacillus* spp. were shown to produce large amounts of 2-butanone [8]. Indeed, 2-butanone is a common VOC secreted from many species of bacteria, fungi, and plants.

In the present study, we aimed to identify effective VOCs with the capacity to elicit SAR in plants. We performed open field application of insect and bacterial volatiles in an attempt to control plant pathogens and insects while minimizing their negative effects on plant growth. Because the bacterial volatile 2,3-butanediol is ineffective in eliciting induced resistance in cucumber plants, our goal was to isolate effective VOCs from among numerous known bacterial volatiles to be used under open field conditions. Here, we demonstrate that VOC-elicited induced resistance can be induced in plants against both a microbial pathogen and an insect pest at the same time. The drench application of 1 mM 3-pentanol and 0.1 μM 2-butanone on cucumber seedlings consistently triggered plant systemic defense responses against *Pseudomonas syringae* pv. lachrymans. Examination of plant defense responses after VOC treatment revealed that the expression of *CsLOX1* was up-regulated. *CsLOX1* encodes a cucumber lypoxygenase, which is a marker protein of the oxylipin pathway. This pathway releases green leaf volatiles (GLVs) to attract natural enemies of pests, thereby protecting the plant against herbivores [29]. During the course of this study, a natural outbreak of aphids occurred. Cucumbers treated with VOCs attracted numerous ladybird beetles, which led to a significant reduction in aphid density, compared with the water control. These results indicate that VOCs can be used to manage plant disease and insect pests by eliciting induced resistance, even in the open field.

## 2. Results and Discussion

### 2.1. 3-Pentanol and 2-Butanone-Elicit Induced Resistance against *Pseudomonas syringae* and Aphids

**Figure 1:**
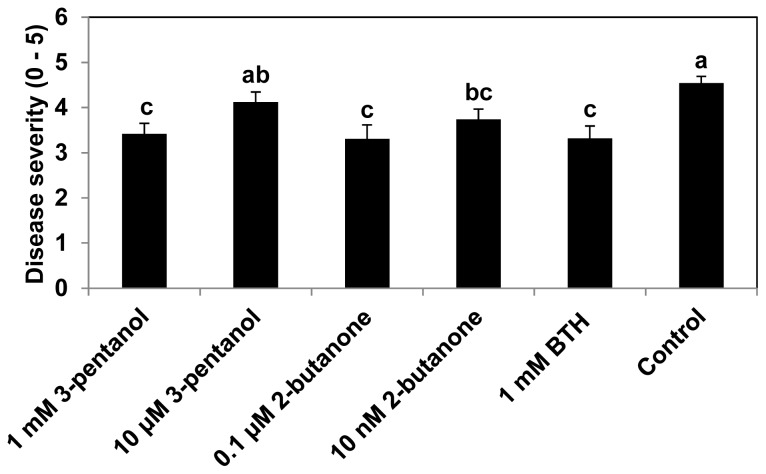
Induction of systemic resistance by 3-pentanol and 2-butanone against *Pseudomonas syringae* pv. lachrymans. The severity of symptoms was scored from 0 to 5 as follows: 0, no symptoms; 1, yellowish color; 2, chlorosis only; 3, partial necrosis and chlorosis; 4, necrosis of the inoculated area and expanded chlorosis; and 5, complete necrosis of the inoculated area. Disease severity of cucumber treated with 3-pentanol and 2-butanone was assessed 7 days after infection with *P. syringae* pv. lachrymans. Water and 0.5 mM benzothiadizole (BTH) were used as negative and positive controls, respectively. Means in columns followed by different letters are significantly different at *P* = 0.05 according to the LSD test. Error bars indicate the standard error (*n* = 16).

Drench application of 3-pentanol and 2-butanone resulted in a reduction in disease severity in cucumber in the open field at 28 days post seeding (dps), *i.e.*, 7 days after spray-challenge of *P. syringae* pv. lachrymans (Figure 1). The treatment of cucumber plants with 1 mM 3-pentanol, 0.1 μM 2-butanone, or 10 nM 2-butanone caused 24%, 26%, or 17% less symptom severity, respectively, than the water control (Figure 1). The disease severity of plants treated with 10 μM 3-pentanol was not statistically different from that of the control (*P* = 0.05). Plants treated with BTH, which was employed as a positive control, showed similar levels of disease severity to plants treated with 0.1 μM 2-butanone.

Among numerous bacterial metabolites, bacterial volatiles have recently been examined for their ability to elicit induced resistance against diverse plant pathogens. For example, the bacterial volatile 2,3-butanediol, produced by *Bacillus* spp. and *P. chlororaphis*, was shown to induce systemic plant defenses against *Pectobacterium carotovorum* subsp. *carotovorum* in *Arabidopsis* and tobacco [6,9]. However, *P. chlororaphis* fails to elicit ISR against *P. syringae* pv. tabaci in tobacco [9]. Thus, the authors of this study concluded that 2,3-butanediol is not effective against biotrophic pathogens, while this compound is effective against necrotrophic pathogens. However, recent reports show that 2,3-butanediol protects *Arabidopsis* seedlings against the biotrophic pathogen *P. syringae* pv. tomato [30]. Therefore, 2,3-butanediol may mediate induced resistance in plants in a species-dependent manner.

Large scale trials of VOCs in the open field often produce inconsistent results, as these results are dependent on the plant species studied and the rapid evaporation of the VOCs after treatment. Field trials examining the effects of 2,3-butanediol treatment on tobacco led to a successful reduction in symptom development [10,11]. Furthermore, we found that 2,3-butanediol is rarely effective at eliciting induced resistance in cucumber plants against the biotrophic pathogen *P. syringae* pv. lachrymans (data not shown). Further screening of VOC-mediated induced resistance against the same pathogen led to the selection of two new resistance-inducing VOC candidates, 3-pentanol and 2-butanone. These volatiles have not previously been shown to elicit induced resistance in any plant species. In the laboratory, root application of 3-pentanol also elicited induced resistance against *Xanthomonas axonopodis* pv. vesicatoria in pepper (unpublished data). The concentration of 3-pentanol required to elicit induced resistance (1 mM) was higher than that of 2-butanone but similar to that of BTH. Similar disease control capacities were observed between 3-pentanol and 1000-fold lower concentrations of 2-butanone (Figure 1). Even at the 10 nM level, the SAR-inducing capacity of 2-butanone was significantly different from that of the control (*p* = 0.05). It is noteworthy that 3-pentanol elicited induced resistance by direct application onto the root as well as by emission of the volatile form under spatially separated conditions between plant and 3-pentanol treatment (unpublished data). To our knowledge, this is the first report of VOC-mediated induced resistance induced by these two compounds in cucumber.

**Figure 2:**
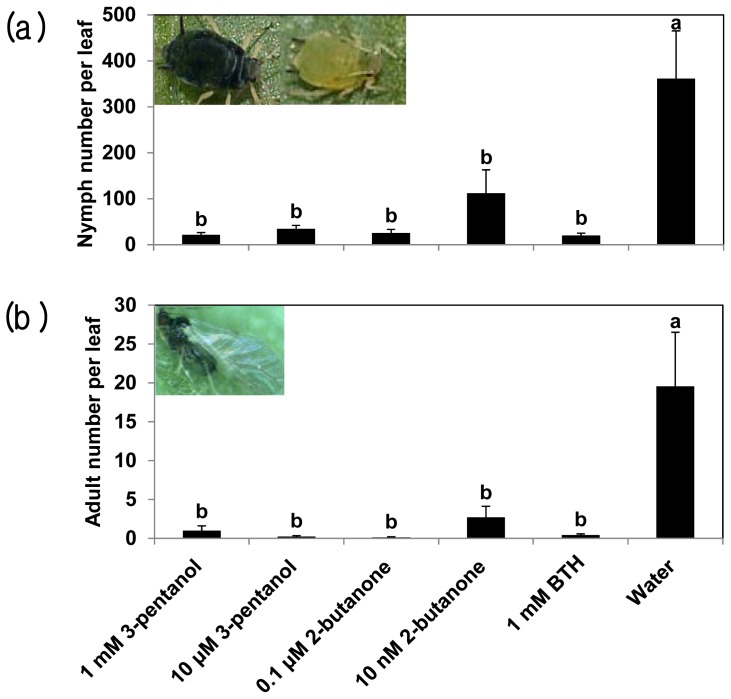
3-Pentanol and 2-butanone confer induced resistance against aphids in cucumber: a, Nymph number; b, Adult number. Bars represent the mean ± SE (sample size, *n* = 12 replications per treatment). Means in columns followed by different letters are significantly different at *P* = 0.05 according to the LSD test.

Unexpectedly, in the mid-summer of 2011, an outbreak of aphids (*Myzus persicae*) occurred in cucumber in the experimental field (Daejeon area, Korea), resulting in severe infestation of the cucumber plants, especially on newly developing leaf tissues. We measured aphid damage by counting the number of aphids (individual nymphs and adults) per leaf. At 34 dps, the number of aphids significantly decreased in all treatments compared with the control. The control plants contained 361 nymphs and 19 adults per leaf. Plants that were soil drenched with 1 mM 3-pentanol, 10 μM 3-pentanol, 0.1 μM 2-butanone, or 10 nM 2-butanone exhibited 21, 34, 25, or 112 nymphs and 1.0, 3.0, 0.1, or 2.7 adults, respectively (Figure 2a,b). Although the detailed mechanisms by which these compounds induce resistance against sucking insects remain to be elucidated, the results were consistent across three repeated trials. As expected, BTH treatment decreased the number of aphids on the plants, with 20 nymphs and 0.4 adult aphids per leaf (Figure 2a,b). Previously, we found that pretreatment of plants with BTH causes a reduction in aphid infestation [31]. An examination of defense-related gene expression demonstrated that SA signaling plays a critical role in this defense response [32]. Treatment with 1 mM 3-pentanol, 10 μM 3-pentanol, or 0.1 μM 2-butanone increased the induced resistance capacity against aphids, with up to 100-fold fewer aphids on treated plants *vs*. the control (Figure 2a,b). Similarly, benzoxazinoid (BX) derivatives were found to modulate plant defenses against the aphid *Rhopalosiphum padi* and the fungal pathogen *Setosphaeria turtica* in a study of BX-deficient bx1 mutant maize plants [33]. The attack of these organisms on wild-type plants resulted in the increased accumulation of 2,4-dihydroxy-7-methoxy-2*H*-1,4-benzoxazin-3(4*H*)-one (DIMBOA), DIMBOA-glucoside, and 2-hydroxy-4,7-dimethoxy-1,4-benzoxazin-3-one-glucoside (HDMBOA-glc), especially in apoplastic leaf extracts, while these compounds did not accumulate in similarly treated bx1 mutant plants [33]. In addition, the level of callose deposition triggered by chitosan, which is a SAR inducer, was significantly lower in the mutant than in the wild type. During the elicitation of SAR in this system, the production of BXs has a direct inhibitory effect on symptom development. Because aphid stylets and bacterial type III secretion systems target the host apoplast before obtaining host nutrients, the increased deposition of toxic BXs in the apoplast is consistent with the role of BXs in blocking the infection process. Due to a lack of biochemical studies in cucumber, we cannot yet determine whether this BX-related mechanism applies to our system. More biochemical studies will be required to understand the function of candidate compounds against both pathogenic bacteria and aphids.

### 2.2. 3-Pentanol and 2-Butanone Treatment Increase the Numbers of Ladybird Beetles

**Figure 3:**
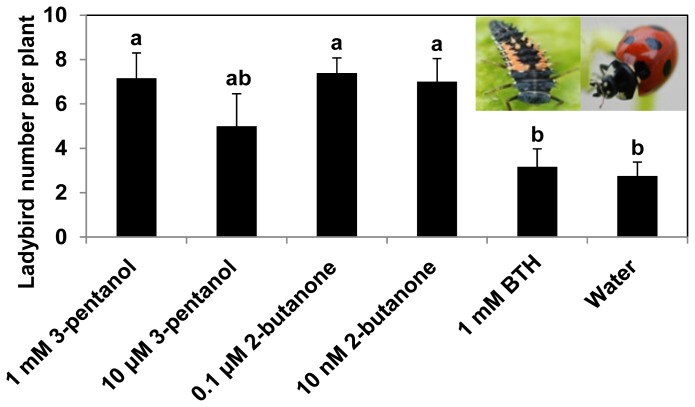
3-Pentanol and 2-butanone treatments increase the number of ladybird beetles. Bars represent the mean ± SE (sample size, *n* = 8 replications per treatment). Means in columns followed by different letters are significantly different at *P* = 0.05 according to the LSD test. The experiment was repeated three times with similar results.

When we were counting the number of aphids on cucumber leaves at each time point, we noticed that the number of spotted ladybird beetles on the leaves differed, depending on the treatment. The mean number of seven spotted ladybird beetles (*Coccinella septempunctata*) on cucumber leaves treated with 1 mM 3-pentanol, 0.1 μM 2-butanone, or 10 nM 2-butanone was 7.2, 7.1 or 7.0, respectively, while the control plant had a mean of 2.8 spotted ladybird beetles per leaf (Figure 3). The two VOC and BTH treatments have statistically significant lower numbers compared to the control treatment. Only treatment with 10 μM 3-pentanol failed to increase the number of ladybird beetles compared with the control (*P* = 0.05). BTH was ineffective at increasing the number of ladybird beetles compared with the control. The ladybird beetle is best known as a natural enemy of insect pests, including aphids. In a closed system such as a greenhouse, and sometimes in open fields, natural enemies have often been used to control insect pests in crop plants [34]. For instance, the predatory generalist ladybird beetle provides biological control of aphids (*Marcosiphulm euphorbiae*) in greenhouse-grown roses [35]. In this system, ladybird beetles successfully reduced the aphid population during an outbreak, without altering the density of a specialist parasitoid, indicating a minimization of ecological disruption by affecting intraguild predation. When we carefully observed parasitoid or spider mite-predator mite dynamics, there were no obvious differences in the insect densities. However, we could not provide a reasonable explanation for the increase in population of ladybird beetles in response to chemical application (except for BTH treatment; Figure 3). Previous data show that aphid-infected plants release volatiles to attract herbivore predators, such as the aphidophagous predators, *Ephisyrphus balteatus*, in potato [36]. This phenomenon, in which the damaged plant emits attractive volatiles to entice beneficial insects, is referred to as “indirect defense” [37]. However, in our system, the aphid numbers were significantly reduced in response to treatment with VOCs or BTH, suggesting that aphid-mediated volatile emission is unlikely to explain the increase in ladybird beetle population. We hypothesize that specific attractive volatiles for ladybird beetles in cucumber plants elicited by 3-pentanol and 2-butanone, but not by BTH, were produced, which recruited the generalist carnivores, resulting in reduced aphid numbers. A detailed analysis of this mechanism would require volatile profiling at different time points after chemical and aphid treatment.

### 2.3. 3-Pentanol and 2-Butanone Do Not Affect Plant Growth but Increase Fruit Yield

**Figure 4:**
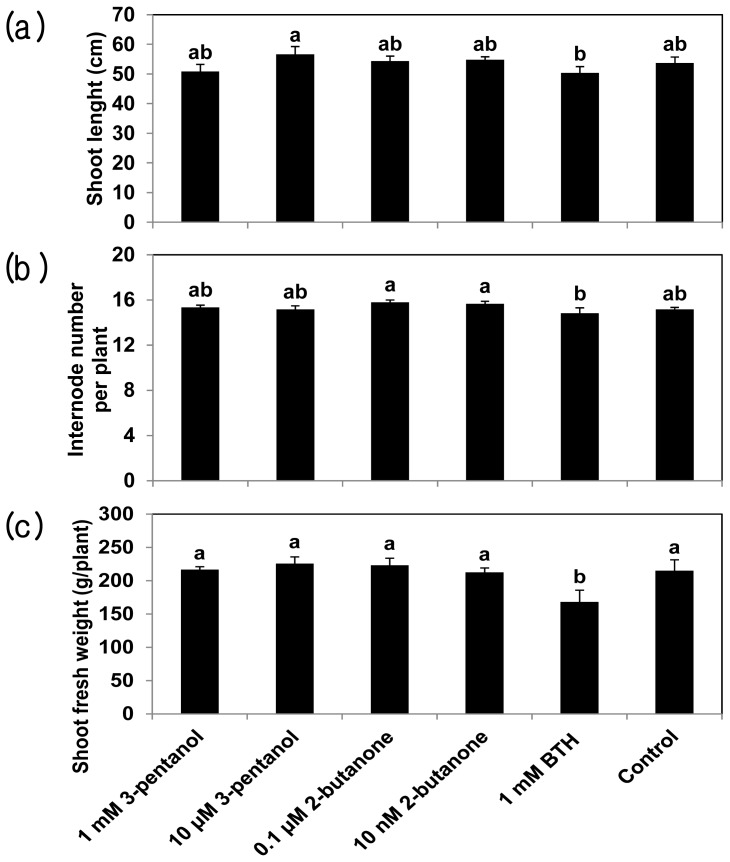
3-Pentanol and 2-butanone do not alter cucumber growth: (**a**) Shoot length; (**b**) Internode number; (**c**) Shoot fresh weight. The growth of 3-pentanol and 2-butanone-treated cucumber plants was assessed at 24 (**a**), 34 (**b**), and 52 (**c**) dps. Water and 1 mM BTH were used as the negative and positive controls, respectively. Bars represent the mean ± SE (sample size, *n* = 8 replications per treatment). Means in columns followed by different letters are significantly different at *P* = 0.05 according to the LSD test. Error bars indicate the standard error (*n* = 8).

To test the effect of two VOCs on plant growth, we employed shoot length, internode number, and shoot fresh weight as growth parameters. The shoot lengths, internode numbers, and shoot fresh weights of the control plants did not differ across the treatments (Figure 4a–c). For shoot length, only 10 μM 3-pentanol produced significant differences in shoot length compared with BTH treatment (Figure 4a). The internode number of plants treated with either concentration of 2-butanone was significantly higher than that of plants treated with 1 mM BTH (Figure 4b). The shoot weights were higher in plants subjected to any of the four induced resistance trigger treatments than in BTH-treated plants (Figure 4c).

**Figure 5:**
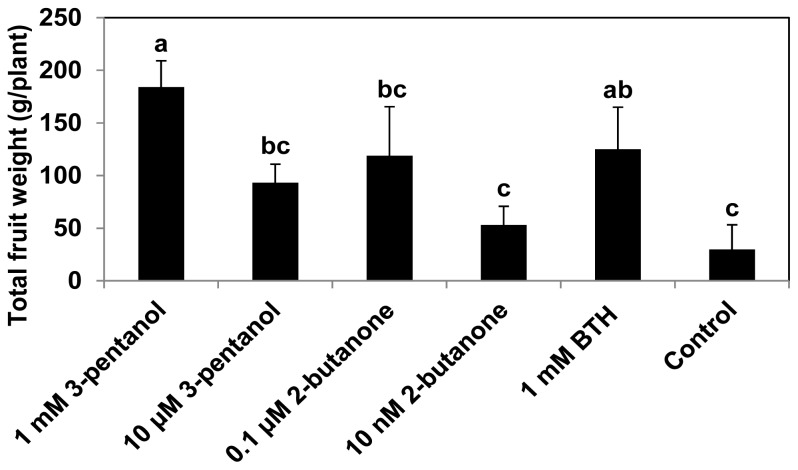
Increase in cucumber yield by 3-pentanol and 2-butanone. Fruit weight of 3-pentanol and 2-butanone-treated cucumber plants was assessed at 52 dps. Bars represent the mean ± SE (sample size, *n* = 8 replications per treatment). Means in columns followed by different letters are significantly different at *P* = 0.05 according to the LSD test. Error bars indicate the standard error.

Previous studies support the notion that overexpression of defense-related genes by stable transformation of plants, and the application of chemical inducers such as BTH, cause significant growth reduction under greenhouse and field conditions [14,15]. BTH-treated pepper exhibits reduced growth in the absence of pathogens [31,38]. This effect may be related to the *de novo* synthesis of harmful compounds, which plays a role in the autotoxicity of plants. To date, no reports have described the identification of chemicals that can elicit induced resistance under field conditions without altering plant growth, although many researchers have struggled to isolate chemicals that induce SAR [39]. In the current study, there was an unexpected promotion of fruit yield in cucumber plants treated with 1 mM 3-pentanol or 0.1 μM 2-butanone as much as 6.13 and 3.96 fold increase respectively compared to control treatment (Figure 5). The mechanism that causes certain concentrations of 3-pentanol and 2-butanone to confer plant protection against aphids and bacterial pathogens while increasing yield is unclear. However, VOC-producing PGPR promote plant growth and systemic defense, even under field conditions. BioYield^®^-containing strains GB03 and IN937a consistently increase the growth and yield of cucumbers, peppers, and tomatoes [4].

The vegetative growth of cucumber was similar in plants treated with 3-pentanol, 2-butanone, or water. The yield of BTH-treated plants did not differ from that of the control. We previously demonstrated that BTH causes significant growth reduction in cucumber in the absence of disease pressure. Indeed, the yield of plants subjected to 3-pentanol or 2-butanone treatment may not have increased as a direct consequence of chemical treatment; this increase may have resulted from a decrease in plant damage caused by pathogens and insects.

### 2.4. Expression of Defense-Related Genes in Response to 3-Pentanol and 2-Butanone

**Figure 6:**
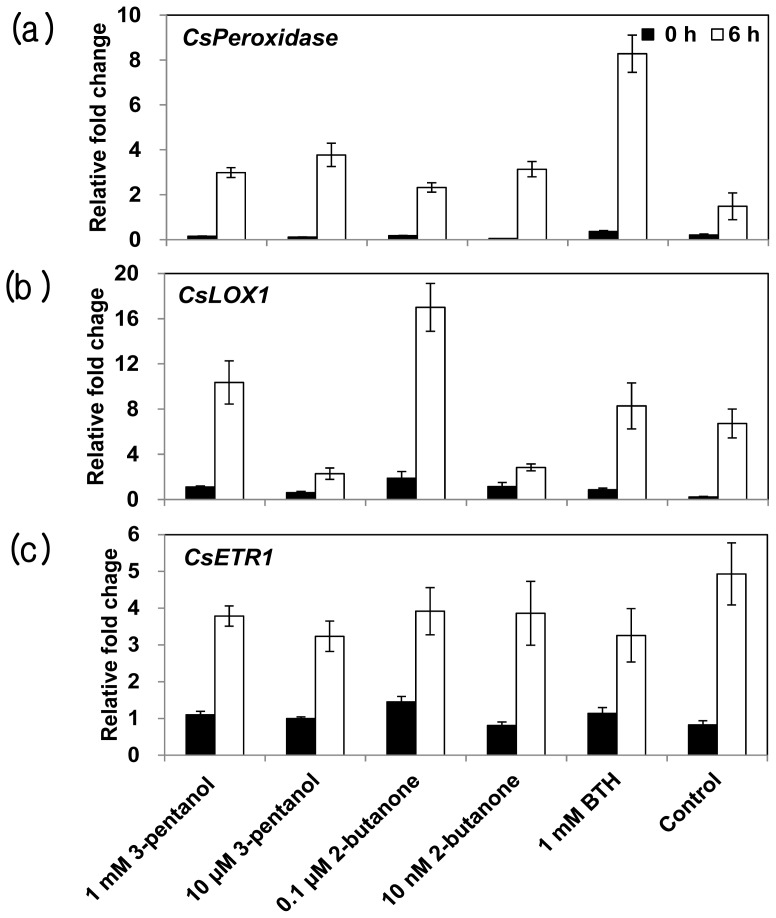
Expression of defense-related genes in response to 3-pentanol and 2-butanone. The expression levels of the cucumber resistance genes Peroxidase, LOX1, and ETR1 were assessed by qRT-PCR at 0 and 6 h after *Pseudomoans syringae* pv. lachrymans challenge in plants pretreated with 3-pentanol or 2-butanone. Bars represent the mean value ± SE (*n* = 3). The housekeeping gene Actin was used as a control. The experiment was repeated twice with similar results.

To decipher the mode of action for SAR signaling, we employed qRT-PCR to investigate defense priming of cucumber defense-related genes including *CsPeroxidase* as SA marker gene [40], *CsLOX1* as JA marker gene [41], and *CsETR1* as ET marker gene [41]. Defense priming is the augmentation of the basal resistance of plants after an initial inoculation by parasites or chemical inducers. Primed plants exhibit quick, strong resistance responses to subsequent pathogen attack [42]. Defense priming represents proficient machinery in the allocation of fitness cost [39]. To measure defense priming, we collected plant samples at 0 and 6 h after spray-challenge of pathogen in cucumber seedlings that had been treated with VOCs or BTH seven days earlier and conducted qRT-PCR analysis. The levels of expression of *CsPeroxidase* and *CsETR1* in plants subjected to either concentration of 3-pentanol or 2-butanone were similar to that of control-treated plants (Figure 6a,c). The *CsLOX1* gene was significantly up-regulated, by as much as 1.5- or 2.5-fold in plants treated with 1 mM pentanol or 0.1 μM butanone, respectively, while this gene was down-regulated by 2.9- or 2.4-fold in plants treated with μM 3-pentanol or 10 nM butanone, respectively (Figure 6b). BTH treatment increased *CaPeroxidase* expression in the systemic cucumber tissue indicating induction of salicylic acid dependent SAR but did not alter *CaLOX1* or *CaETR1* expression which indicates JA and ET signaling pathways corresponding to normal ISR. To our knowledge, the role of peroxidase has not been extensively studied in cucumber compared to that in *Arabidopsis*. It will be good topic to study the detail function of peroxidase in cucumber with insect or pathogen attack.

When insects feed on plant tissues, anti-herbivore defense responses are induced in the plant. These defenses function either directly, through the production of anti-digestive protein or toxic/repelling chemicals [43], or indirectly via emission of VOCs, known as GLVs, which recruit natural enemies of the target herbivores [29]. The VOCs are produced by the activation of the plant oxylipin pathway, which produces these compounds from the oxygenated derivatives of fatty acids in plant cell membranes [44]. LOX converts linolenate (derived from membrane lipids via lipases) to hydroperoxy linolenate, which is a source for the production of the major GLVs 3-hexenal, 3-hexanol, and 3-hexyl acetate, as well as jasmonate [45]. We did not examine the levels and profiles of such GLVs in response to aphid sucking or chemical treatment. In a future study, we plan to obtain these GLV profiles and investigate the attraction of ladybird beetles to aphid-infested cucumber plants treated with 3-pentanol and 2-butanone. Kappers *et al*. (2010) reported that the attack of arthropods on cucumber releases VOCs, which in turn attract carnivorous insects [46]. A greenhouse study using the herbivorous spider mites *Tetranychus urticae* and its predatory mite *Phytoseiulus persimilis* revealed that plants emit (*E*)-β-ocimene, (*E*,*E*)-4,8,12-trimethyl-1,3,7,11-tridecatetraene ([*E*,*E*]-TMTT), and two unidentified compounds that are positively correlated to the attraction of the spider mite’s natural enemy. The elicitation of VOCs can be induced by exposing plants to the plant hormone jasmonic acid. A total of 24 VOCs, mainly terpenoids, were strongly emitted by cucumber plants in response to jasmonic acid treatment rather than by spider mite infestation or other chemical treatments. The release of these VOCs is correlated with the attraction of predatory insects, indicating that jasmonic acid-mediated plant VOCs play an important role in indirect defenses in cucumber. An investigation that combined transcriptome analysis and volatile profiling to elucidate the possible involvement of target genes in the synthesis of these volatiles, provided evidence for the production of (*Z*)-3-hexenyl acetate through the activity of lipoxygenase genes [47]. It is worth noting that aphid infestation elicited the expression of the jasmonic acid signaling-related gene *CsLOX1* but did not alter the expression of SA signaling-related genes, depending on 3-pentanol and 2-butanone treatments. Normally, insects with piercing-sucking mouthparts trigger the expression of more SA-related defense genes (such as PR genes) than chewing insects, and these genes induce the expression of JA-related defense genes [48]. Perhaps cross-talk between SA and JA signaling induced by pretreatment with 3-pentanol or 2-butanone leads to the induction of the JA signaling pathway, as indicated by the increased expression of *CsLOX1*. Taken together, this study suggests that the application of 3-pentanol or 2-butanone triggers the expression of oxylipin pathway genes in cucumber, resulting in the emission of GLVs, which act as attractants to natural enemies of aphids, such as ladybird beetles.

## 3. Experimental Section

### 3.1. Plant Growth and Chemical Treatment

Cucumber plants (*Cucumis sativus* L. cv. backdadagi) were cultivated in an open field under natural conditions. For greenhouse experiments, seeds were directly planted in pots containing soilless medium (Punong Co. Ltd., Gyeongju, Korea) in a 24 hole plug tray under greenhouse condition. The germinated seedlings were transplanted into large pots (diameter = 30 cm; height = 30 cm). Chemical treatments to elicit induced resistance in cucumber were carried out as previously described [49]. Briefly, the 14 day old cucumber seedlings were treated by direct drench application of 50 mL solution of 1 mM 3-pentanol, 10 μM 3-pentanol, 0.1 μM 2-butanone, or 10 nM 2-butanone. All chemicals, bought from Sigma-Aldrich Co. were dissolved in distilled water before application. Treatments with 0.5 mM benzothiadiazole (BTH) and water were used as positive and negative controls, respectively.

### 3.2. Assessment of Angular Leaf Spot Disease and Aphid Infestation

For pathogen challenge, a culture of the compatible bacterial pathogen *Pseudomonas syringae* pv. lachrymans (OD_600_ = 1 in 10 mM MgCl_2_) was spray-challenged on cucumber leaves until run-off at 7 days after the drench application of chemicals to roots at 21 days after seeding. The severity of symptoms was scored from 0 to 5 as follows: 0, no symptoms; 1, less than 20% diseased area; 2, 21%–40% diseased area; 3, 41%–60% diseased area; 4, 61%–80% diseased area; and 5, more than 81% diseased area of the entire leaf. Bacterial pathogens were cultured overnight at 28 °C in King’s B medium supplemented with the appropriate antibiotics (100 μg/mL). As a positive control, roots were treated with 0.5 mM BTH. Leaves were harvested at the indicated times and frozen immediately in liquid nitrogen for total RNA extraction. Intact cucumber leaves were used for non-stress treatments. Following inoculation with the pathogen, leaf tissue was harvested 0 and 6 h post-inoculation (hpi) prior to the isolation of total RNA. The experiment had a completely randomized design, with ten replications, and was independently repeated four times. To investigate whether the two VOCs elicited plant immunity to aphid feeding, numbers of naturally occurring aphids (in 2011, Daejeon, Korea) were counted. Application of 0.5 mM BTH was used as a positive control. The total numbers of nymph and adult aphids were counted at 34 days after seeding. The experiments were repeated with similar results.

### 3.3. Measurement of Plant Growth Parameters

To assess the effects of both VOCs on plant growth under insect and disease pressure, growth parameters, including shoot length, internode number, and shoot fresh weight were measured at 24, 34, and 44 dps, respectively. Additionally, to assess the effect of 3-pentanol and 2-butanone on fruit yields, fruits were harvested at 34 dps. The yields, *i.e.*, total fruit weight per plant, were calculated, and the experiment was repeated three times.

### 3.4. RT-PCR and Quantitative RT-PCR

Real-time PCR was performed using a Bio-Rad CFX96 system. Total RNA was isolated from cucumber leaf tissues using Tri reagent (Molecular Research Inc. Cincinnati, OH, USA) according to the manufacturer’s instructions. First-strand cDNA synthesis was carried out with 2 μg of DNase-treated total RNA, oligo-dT primers, and Moloney murine leukemia virus reverse transcriptase (MMLV-RT, Enzynomics, Daejeon, Korea). PCR reactions were carried out according to the manufacturer’s instructions. The expression of candidate priming gene was analyzed using the following primers: 5′-AGAGCAACAAGGTCGGTTTCA-3′ (*Csperoxidase*-F), 5′-GTGCCGACATCC TAGCTCAAG-3′ (*Csperoxidase*-R), 5′-AAGGTTTGCCTGTCCCAAGA-3′ (*CsLOX1*-F), 5′-TGAG TACTGGATTAACTCCAGCCAA-3′ (*CsLOX1*-R), 5′-GCCATTGTTGCAAAAGCAGA-3′ (*CsETR1*-F), 5′-GCCAAAGACCACTGCCAC-3′. As a control to ensure that equal amounts of RNA were analyzed in each experiment, the expression of *CsActin* was analyzed using the primers 5′-CCGT TCTGTCCCTCTACGCTAGTG-3′ and 5′-GGAACTGCTCTTTGCAGTCTCGAG-3′ [50]. Candidate genes were amplified from 100 ng of cDNA by PCR using an annealing temperature of 55 °C. A Chromo4 real-time PCR system (BIO-RAD Inc., Hercules, CA, USA) was used to carry out quantitative RT-PCR. Reaction mixtures consisted of cDNA, iQTM SYBR® Green Supermix (BIO-RAD Inc.), and 10 pM of each primer. The thermocycler parameters were as follows: initial polymerase activation, 10 min at 95 °C followed by 40 cycles of 30 s at 95 °C, 60 s at 55 °C, and 30 s at 72 °C. The conditions were determined by comparing threshold values in a series of dilutions of the RT product, followed by a non-RT template control and a non-template control for each primer pair. The relative RNA quantification, calculated using the 2^−ΔΔCT^ method, together with standard errors of mean values among replicates, was conducted using Bio-Rad Manager (version 2.1; Bio-Rad CFX Connect, Hercules, CA, USA). A Student’s *t*-test was carried out to determine statistically significant differences between treated and untreated samples. If *p*-values <0.05, then the target genes were considered to be differentially expressed. Relative RNA levels were calibrated and normalized against expression levels of *CsActin* mRNA (GenBank accession no. AB010922).

### 3.5. Statistical Analysis

Analysis of variance for experimental datasets was performed using JMP software (version 5.0; SAS Institute, Inc., Cary, NC, USA). Significant effects of treatment were determined by the magnitude of the *F* value (*P* = 0.05). When a significant *F* test was obtained, separation of means was accomplished by Fisher’s protected LSD at *P* = 0.05.

## 4. Conclusions

To overcome the disadvantage of VOC application to agricultural fields, *i.e.*, the rapid evaporation rates of VOCs, we determined whether the application of two VOCs, 3-pentanol and 2-butanone, would elicit induced resistance against a microbial pathogen. The results demonstrated that the two VOCs successfully protected cucumber plants against the biotrophic bacterial pathogen *P. syringae* in open field trials. Unexpectedly, these VOCs caused a significant reduction in aphid numbers, as well as an increased number of the natural enemy of aphids, the ladybird beetle. Upregulation of *CsLOX1* expression (a gene involved in an early step of the oxylipin pathway) in cucumber plants pretreated with 3-pentanaol or 2-butanone indicated the induction of indirect defenses. The plants emitted alarm volatile compounds, such as GLVs, which attract the natural enemy of aphids. This study may lead to the development of new ways to apply VOCs to manage microbial pathogens and herbivores under open field conditions.
